# BLT_1_ signalling protects the liver against acetaminophen hepatotoxicity by preventing excessive accumulation of hepatic neutrophils

**DOI:** 10.1038/srep29650

**Published:** 2016-07-11

**Authors:** Ken Kojo, Yoshiya Ito, Koji Eshima, Nobuyuki Nishizawa, Hirotoki Ohkubo, Takehiko Yokomizo, Takao Shimizu, Masahiko Watanabe, Masataka Majima

**Affiliations:** 1 Departments of Pharmacology, Kitasato University School of Medicine, 1-15-1 Kitasato, Minami, Sagamihara, Kanagawa, 252-0374, Japan; 2 Departments of Surgery, Kitasato University School of Medicine, 1-15-1 Kitasato, Minami, Sagamihara, Kanagawa 252-0374, Japan; 3 Departments of Immunology Kitasato University School of Medicine, 1-15-1 Kitasato, Minami, Sagamihara, Kanagawa 252-0374, Japan; 4 Departments of Cardiovascular Surgery, Kitasato University School of Medicine, 1-15-1 Kitasato, Minami, Sagamihara, Kanagawa 252-0374, Japan; 5 Department of Biochemistry, Juntendo University School of Medicine, 2-1-1 Hongo, Bunkyo-ku, Tokyo 113-8421, Japan; 6 Department of Lipid Signaling, National Center for Global Health and Medicine, 1-21-1 Toyama, Shinjuku-ku, Tokyo 162-0052, Japan

## Abstract

Leukotriene B_4_ (LTB_4_) is a potent chemoattractant for neutrophils. Signalling of LTB_4_ receptor type 1 (BLT_1_) has pro-inflammatory functions through neutrophil recruitment. In this study, we investigated whether BLT_1_ signalling plays a role in acetaminophen (APAP)-induced liver injury by affecting inflammatory responses including the accumulation of hepatic neutrophils. BLT_1_-knockout (BLT_1_^−/−^) mice and their wild-type (WT) counterparts were subjected to a single APAP overdose (300 mg/kg), and various parameters compared within 24 h after treatment. Compared with WT mice, BLT_1_^−/−^ mice exhibited exacerbation of APAP-induced liver injury as evidenced by enhancement of alanine aminotransferase level, necrotic area, hepatic neutrophil accumulation, and expression of cytokines and chemokines. WT mice co-treated with APAP and ONO-0457, a specific antagonist for BLT_1_, displayed amplification of the injury, and similar results to those observed in BLT_1_^−/−^ mice. Hepatic neutrophils in BLT_1_^−/−^ mice during APAP hepatotoxicity showed increases in the production of reactive oxygen species and matrix metalloproteinase-9. Administration of isolated BLT_1_-deficient neutrophils into WT mice aggravated the liver injury elicited by APAP. These results demonstrate that BLT_1_ signalling dampens the progression of APAP hepatotoxicity through inhibiting an excessive accumulation of activated neutrophils. The development of a specific agonist for BLT_1_ could be useful for the prevention of APAP hepatotoxicity.

Acetaminophen (N-acetyl-para-aminophenol) (APAP) hepatotoxicity from overdose can result in severe hepatic damage, which is characterised by haemorrhagic centrilobular necrosis and high plasma levels of transaminases in both humans and animals^1^. Formation of the main reactive metabolite of APAP (N-acetyl-p-benzoquinone imine; NAPQI), which results from the metabolic activation of APAP by the cytochrome P450 family (principally CYP2E1; CYP3A4), is an important step in the development of liver injury^2^. NAPQI causes glutathione (GSH) depletion and covalent cellular protein modifications, resulting in mitochondrial dysfunction and oncotic necrosis^3^. The intracellular signalling pathways leading to oncotic necrosis after APAP overdose are critical for the mechanisms underlying APAP hepatotoxicity.

In addition to direct hepatocellular damage through metabolic activation of APAP, the subsequent innate immune responses have been considered to modulate liver injury elicited by APAP overdose^4^,^5^. However, it is currently uncertain whether accumulation of innate immune cells, particularly neutrophils, plays a role in the injury elicited by APAP^6^,^7^,^8^,^9^. Other cell types of the innate immune system, such as macrophages/monocytes, appear to mediate the resolution and repair of liver damage through the clearance of cellular debris and the promotion of tissue regeneration during APAP hepatotoxicity^10^,^11^,^12^. Pro-inflammatory mediators generated from innate immune cells together with damaged hepatocytes are important for APAP hepatotoxicity^4^,^5^. Prostanoids including prostaglandins (PGs) are metabolites of arachidonic acid formed via the cyclooxygenase (COX) pathway. PGE_2_ has a hepatoprotective effect on APAP toxicity in zebrafish^13^, and COX-2 protects the liver from APAP hepatotoxicity in mice^14^.

Leukotrienes (LTs) are also metabolites of arachidonic acid formed via the 5-lipoxygenase (5-LOX) pathway^15^,^16^. LTB_4_ is a potent chemoattractant for leukocytes, particularly granulocytes^17^,^18^,^19^, and is an important mediator of inflammation^20^,^21^. LTB_4_ binds to two distinct receptors, which are cell surface G-protein-coupled receptors, namely, LTB_4_ receptor type 1 (BLT_1_), a high-affinity LTB_4_ receptor, and BLT_2_, a low-affinity LTB_4_ receptor^20^,^22^,^23^. The potent biological effects of LTB_4_ are mediated primarily through BLT_1_, which is expressed preferentially in neutrophils and monocytes/macrophages^19^,^24^,^25^. BLT_2_ is ubiquitously expressed by various cell types. BLT_1_ signalling plays a pivotal role in several inflammatory diseases through the recruitment of neutrophils into inflammatory sites^26^,^27^,^28^.

However, it remains unknown whether BLT_1_ signalling contributes to liver injury elicited by APAP. Therefore, in the present study, we investigated whether BLT_1_ signalling plays a role in APAP-induced liver injury by affecting inflammatory responses including the accumulation of hepatic neutrophils.

## Results

### BLT_1_ signalling deficiency exacerbates APAP-induced liver injury

**Figure 1:**
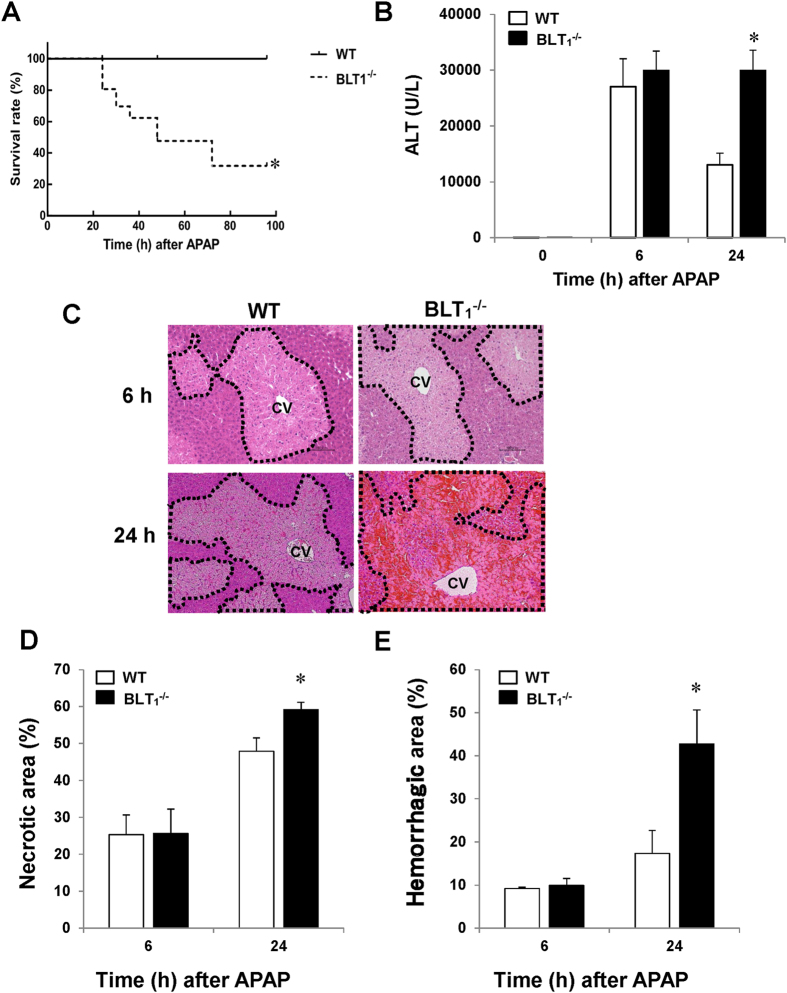
BLT_1_ signalling deficiency exacerbates APAP-induced mortality and liver injury in mice. BLT_1_^−/−^ mice and their WT littermates were administered APAP (300 mg/kg) by i.p. injection. (**A**) The survival rates of BLT_1_^−/−^ mice (n = 20) and WT mice (n = 20) at various time points after receiving APAP. (**B**) Serum levels of ALT after APAP administration. Data are expressed as the mean ± SEM of six mice per group. *p < 0.05 vs. WT mice. Serum samples at 0, 6 and 24 h after APAP administration were collected for measurement of ALT levels. (**C**) Typical appearance of liver tissues stained with H&E in WT and BLT_1_^−/−^ mice at 6 and 24 h after APAP injection (scale bar = 100 μm). The area surrounded by the black dotted line indicates the centrilobular necrotic area. CV, central vein. (**D**) The percentage of the hepatic necrotic area after APAP administration. Data are expressed as the mean ± SEM of six mice per group. *p < 0.05 vs. WT mice. (**E**) The percentage of the haemorrhagic necrotic area after APAP administration. Data are expressed as the mean ± SEM of six mice per group. *p < 0.05 vs. WT mice.

To explore the role of BLT_1_ signalling in APAP hepatotoxicity, BLT_1_-deficient (BLT_1_^−/−^) and wild-type (WT) mice were treated with 300 mg/kg APAP by intraperitoneal (i.p.) injection. We first investigated the impact of BLT_1_ signalling deficiency on APAP-induced mortality in both BLT_1_^−/−^ and WT mice, followed by monitoring the survival rate of the mice every 12 h until 96 h after drug administration. The survival rate of APAP-treated BLT_1_^−/−^ mice was significantly lower than that of APAP-treated WT mice throughout the observation period (Fig. 1A). At 96 h after APAP injection, all WT mice remained alive, whereas the survival rate of BLT_1_^−/−^ mice was 32.4%. Taken together, these findings suggest that disruption of BLT_1_ signalling renders mice more susceptible to APAP hepatotoxicity and mortality. Because more than half of BLT_1_^−/−^ mice were dead within 48 h after APAP administration, we compared WT and BLT_1_^−/−^ mice within 24 h after APAP treatment in subsequent experiments.

We next examined the relative role of BLT_1_ signalling in APAP hepatotoxicity by performing biochemical and histological evaluation of the liver injury. The serum level of aminotransferase (ALT) was markedly elevated in WT mice at 6 and 24 h after APAP treatment (Fig. 1B). By contrast, the ALT level at 24 h in BLT_1_^−/−^ mice sustained high (2.3-fold increase) in comparison with that in WT mice. Histological analysis also revealed the presence of typical centrilobular necrosis in WT mice after APAP administration (Fig. 1C). The hepatic necrotic area was more extensive (23% increase) in BLT_1_^−/−^ mice than in WT mice (Fig. 1D). These results indicate that BLT_1_^−/−^ mice displayed sustained liver injury elicited by APAP, and that BLT_1_ signalling plays an important role in APAP hepatotoxicity. In addition, substantial hepatic haemorrhagic necrosis at 24 h was observed in BLT_1_^−/−^ mice, but not in WT mice (Fig. 1C). Indeed, quantitative analysis revealed that BLT_1_^−/−^ mice had a 2.5-fold larger area of hepatic haemorrhage at 24 h than WT mice (Fig. 1E). These results suggest that the endogenous LTB_4_/BLT_1_ signalling pathway is essential for protecting liver sinusoidal endothelial cells (LSECs) from injury in response to APAP.

### BLT_1_ signalling deficiency enhances the accumulation of hepatic neutrophils after APAP treatment

**Figure 2:**
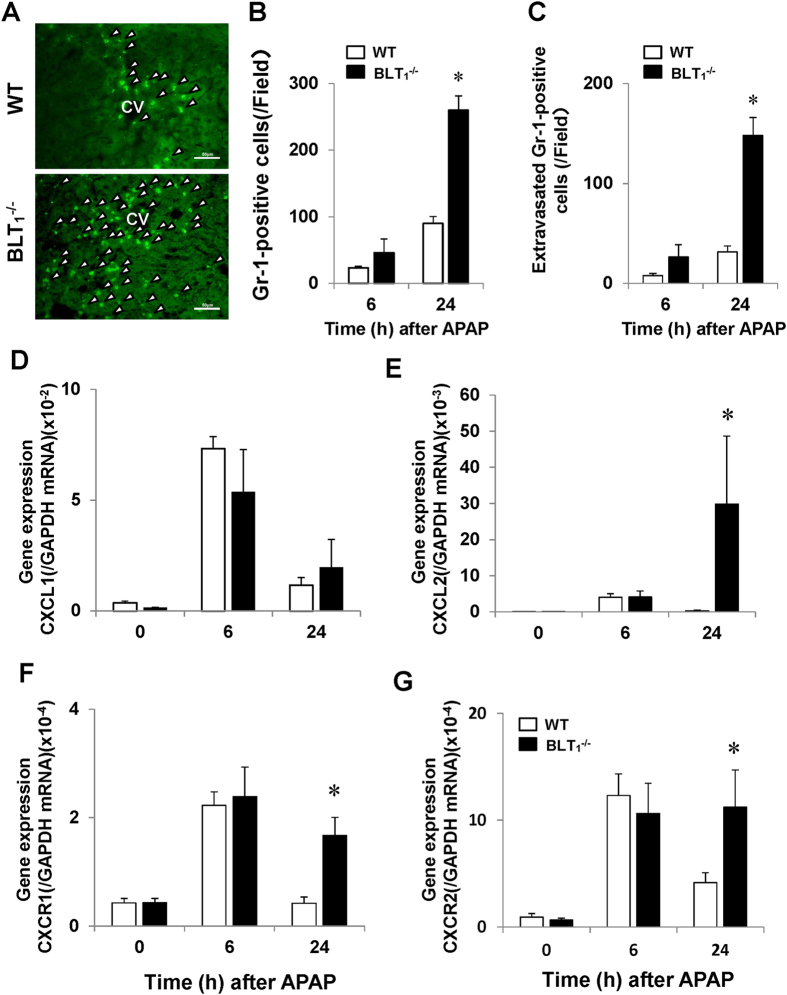
Hepatic neutrophils and hepatic mRNA expression levels of chemokines and their receptors in WT and BLT_1_^−/−^ mice during APAP hepatotoxicity. (**A**) Representative images of liver sections in immunostaining analysis with an anti- Gr-1 antibody in WT mice (upper panel) and BLT_1_^−/−^ mice (lower panel) at 24 h after APAP administration. Arrow heads indicate Gr-1-positive cells. CV, central vein. Bars = 50 μm. (**B**) Changes in the total numbers of cells immunopositive for Gr-1 (neutrophils) in the livers of WT and BLT_1_^−/−^ mice after APAP administration. Data are expressed as the means ± SEM of 5–6 mice per group. *p < 0.05 vs. WT mice. (**C**) Changes in the numbers of extra-sinusoidal neutrophils in the livers of WT and BLT_1_^−/−^ mice after APAP administration. Data are expressed as the means ± SEM of 5–6 mice per group. *p < 0.05 vs. WT mice. (**D–G**) Hepatic mRNA expression levels of chemokines and their receptors, including CXCL1 (**E**), CXCL2 (**F**), CXCR1 (**G**) and CXCR2 (**H**), in WT and BLT_1_^−/−^ mice after APAP administration. Data are expressed as the means ± SEM of 5–6 mice per group. *p < 0.05 vs. WT mice.

Because BLT_1_ signalling is essential for the recruitment of neutrophils into inflamed tissues, we determined the numbers of accumulated neutrophils in the liver during APAP hepatotoxicity. Immunofluorescence staining analysis with an antibody specific for the neutrophil marker, Gr-1, revealed fewer hepatic Gr-1-positive cells in both WT and BLT_1_^−/−^ mice at 0 h (data not shown). In WT mice, hepatic neutrophils accumulated into the necrotic area over time after APAP administration (Fig. 2A). Most Gr-1-positive cells were located in the injured lesions (Figs 2A and S1A). Unexpectedly, the accumulation of hepatic neutrophils in BLT_1_^−/−^ mice was extensive (Fig. 2A). Quantitative analysis revealed that the number of recruited hepatic neutrophils was 2.9-fold higher in BLT_1_^−/−^ mice than in WT mice (Fig. 2B). The number of extra-sinusoidal Gr-1-positive cells at 24 h was higher (4.7-fold increase) in BLT_1_^−/−^ mice than in WT mice (Figs 2C and S1B). Approximately 60% of neutrophils were situated outside of sinusoids.

Neutrophils accumulated into the livers of BLT_1_^−/−^ mice as well as WT mice (Fig. 2A), and we determined the levels of chemokine/chemokine receptors during APAP hepatotoxicity. RT-PCR analysis demonstrated that the hepatic level of chemokine (C-X-C motif) ligand 2 (CXCL2), but not of CXCL1, was higher in BLT_1_^−/−^ mice than in WT mice (Fig. 2D,E). Additionally, the hepatic levels of CXC receptor 1 (CXCR1) and CXCR2 at 24 h were up-regulated in BLT_1_^−/−^ mice in comparison with WT mice (Fig. 2F,G).

### The BLT_1_ receptor antagonist ONO-4057 magnifies APAP-induced liver injury

**Figure 3:**
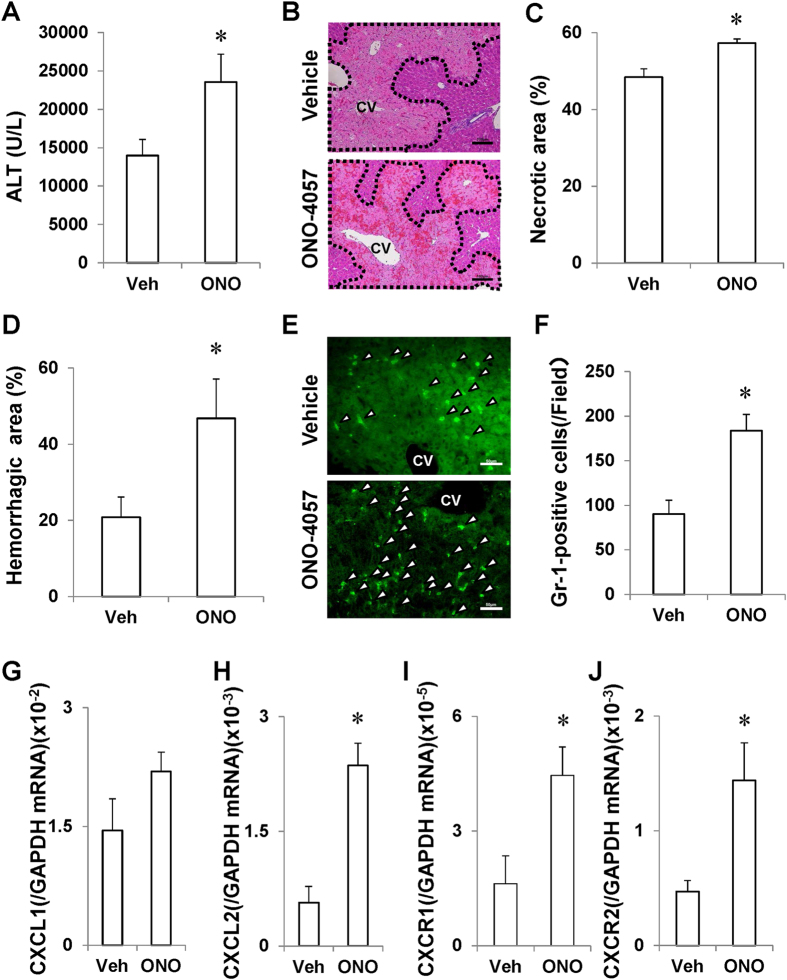
Pharmacological inhibition of BLT_1_ with ONO-4057 exacerbates APAP-induced liver injury in WT mice. (**A–C**) Treatment of WT mice with a BLT_1_ antagonist, ONO-4057, aggravated APAP-induced liver injury at 24 h post-treatment, as evidenced by an increased ALT level (**A**), a larger necrotic area (**B,C**) and a larger haemorrhagic area (**D**) in comparison with WT mice treated with vehicle (Veh). (**B**) Typical images of H&E staining of livers in vehicle-treated mice (upper panel) and ONO-0457-treated mice (lower panel) at 24 h after APAP injection. Necrotic area is delineated with the black dashed line. CV, central vein. Bars = 100 μm. ONO, ONO-4057. Data are expressed as the means ± SEM of 5–6 mice per group. *p < 0.05 vs. vehicle (Veh)-treated WT mice. (**E**) Typical images of immunostaining with an anti-Gr-1 antibody in livers of vehicle-treated mice (upper panel) and ONO-0457-treated mice (lower panel) at 24 h after APAP injection. Arrow heads indicate Gr-1-positive cells. CV, central vein. Bars = 50 μm. (**F,G**) Treatment of WT mice with ONO-0457 increased the recruitment of Gr-1-positive cells into the liver (**E,F**) and increased the mRNA levels of chemokines and their receptors including CXCL2 (**H**), CXCR1 (**I**) and CXCR2 (**J**), but not CXCL1 (**G**), at 24 h after APAP administration. Data are expressed as the means ± SEM of 5–6 mice per group. *p < 0.05 vs. vehicle (Veh)-treated WT mice. ONO, ONO-4057.

To further validate the role of BLT_1_ in APAP hepatotoxicity, WT mice were treated with a BLT_1_ receptor antagonist, ONO-4057, concomitantly with APAP administration. ONO-4057 exacerbated APAP-induced liver injury as evidenced by elevation of the ALT level (1.7-fold), necrotic area (18%) and haemorrhagic area (2.6-fold) in comparison with vehicle treatment (Fig. 3A–D). Compared with that in the livers of vehicle-treated mice, the number of accumulated Gr-1-positive cells in the livers of ONO-0457-treated mice was increased by 2.0-fold (Fig. 3E,F), which was associated with enhanced mRNA hepatic levels of CXCL2, CXCR1 and CXCR2 (Fig. 3G–J). These findings indicated that pharmacological inhibition of BLT_1_ signalling as well as deletion of the BLT_1_ gene exacerbates APAP-induced liver injury.

### Induction of BLT_1_ and 5-LOX expression during APAP hepatotoxicity

The level of BLT_1_ mRNA expression in the livers of WT mice was increased at 6 and 24 h after APAP administration by 2.6- and 2.4-fold, respectively (Fig. S2A). There were no differences in the level of BLT_2_ between the groups (Fig. S2B). The hepatic 5-LOX level in WT mice was also elevated, with a peak at 6 h (an approximately 4-fold increase), and gradually reduced at 24 h (Fig. S2C). BLT_1_^−/−^ mice exhibited similar changes to WT mice at 6 h. The 5-LOX level in the livers of BLT_1_^−/−^ mice remained high at 24 h, but there was no difference between the phenotypes.

To further examine the cellular source of LTB_4_ during APAP hepatotoxicity, fluorescein immunostaining for 5-LOX was performed. Immunofluorescence staining of liver sections of WT mice with an antibody against 5-LOX labelled sinusoids and injured hepatocytes (Fig. S2D). Immunofluorescence double staining of liver sections of WT mice with antibodies against 5-LOX and CD31, CD68, Gr-1 or albumin revealed that 5-LOX in WT livers was expressed mainly in LSECs (CD31-positive cells) (Fig. S2E) and injured hepatocytes (albumin-positive cells) (Fig. S1F), and to a lesser extent in macrophages (CD68-positive cells) (Fig. S2G) and neutrophils (Gr-1-positive cells) (Fig. S2H). These results suggest that LTB_4_ is derived mainly from LSECs and hepatocytes, and partly from macrophages and neutrophils.

### Disruption of BLT_1_ signalling does not affect APAP metabolism

To rule out the possibility that exacerbated liver injury in BLT_1_^−/−^ mice resulted from an altered capacity to metabolically activate APAP, we examined the time course of hepatic CYP2E1 mRNA expression. Hepatic expression of CYP2E1, which is involved in APAP bioactivation, did not differ between the groups (Fig. S3A). APAP overdose triggers liver injury by depletion of intracellular GSH, which in turn causes oxidative stress. We next compared the level of GSH between APAP-treated BLT_1_^−/−^ and WT mice. The hepatic GSH concentration was significantly decreased in both BLT_1_^−/−^ and WT mice after APAP treatment (Fig. S3B). However, there was no significant difference in the hepatic GSH concentration between the two groups of mice. The same was true for the glutathione disulphide (GSSG) level (Fig. S3C) and the ratio of GSH to GSSG (Fig. S3D). These results suggest that both groups had a similar capacity to metabolically activate APAP and that impaired BLT_1_ signalling does not affect APAP metabolism.

### Pro-inflammatory mediators during APAP hepatotoxicity

**Figure 4:**
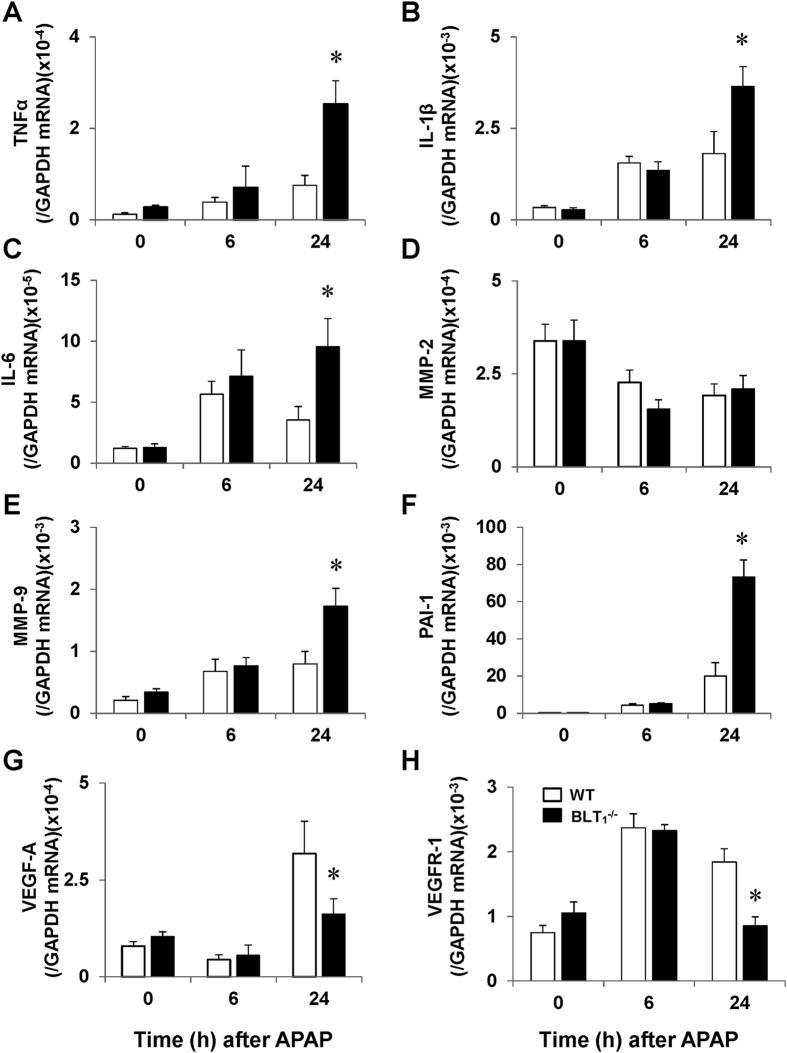
Hepatic mRNA expression levels of pro-inflammatory mediators in WT and BLT_1_^−/−^ mice after APAP administration. (**A–F**) The mRNA hepatic levels of TNFα (**A**), IL-1β (**B**), IL-6 (**C**), MMP-2 (**D**), MMP-9 (**E**), PAI-1 (**F**), VEGF-A (**G**) and VEGFR1 (**H**) in the livers of WT and BLT_1_^−/−^ mice after APAP administration. Data are expressed as the means ± SEM of 3–4 mice per group. *p < 0.05 vs. WT mice.

To investigate whether extensive parenchymal injury in APAP-treated BLT_1_^−/−^ mice is associated with increases in pro-inflammatory mediators, we determined the hepatic mRNA levels of cytokines. Compared with those in WT mice, the hepatic mRNA levels of tumour necrosis factor-α (TNFα), interleukin (IL)-1β and IL-6 in BLT_1_^−/−^ mice were increased by 3.4-fold, 2.0-fold and 2.7-fold, respectively (Fig. 4A–C). With respect of sinusoidal injury in response to APAP, we determined the mRNA levels of matrix metalloproteinase (MMP)-2, MMP-9 and plasminogen activator inhibitor (PAI)-1 (Fig. 4D–F). The levels of MMP-9 (2.2-fold) and PAI-1 (3.7-fold), but not of MMP-2, were elevated in the livers of BLT_1_^−/−^ mice. We also measured the levels of vascular endothelial growth factor (VEGF)-A and VEGF receptor 1 (VEGFR1) because VEGF/VEGFR1 signalling is involved in sinusoidal injury during APAP hepatotoxicity^11^. Hepatic expression of VEGF-A and VEGFR1 in BLT_1_^−/−^ mice was down-regulated by approximately 50% (Fig. 4G,H).

### Hepatic neutrophil activation in BLT_1_^−/−^ mice during APAP hepatotoxicity

**Figure 5:**
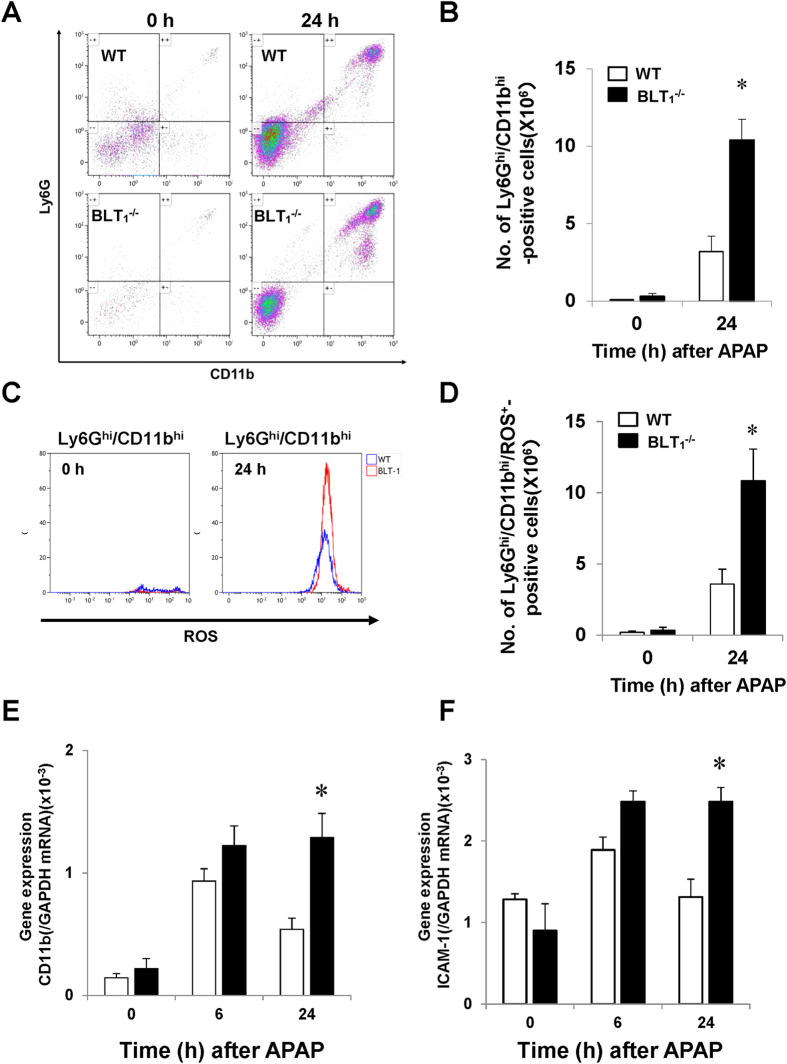
Hepatic neutrophils in WT and BLT_1_^−/−^ mice during APAP hepatotoxicity. (**A**) Representative flow cytometry plots for Ly6G^hi^/CD11b^hi^ cells after APAP administration. (**B**) Population of Ly6G^hi^/CD11b^hi^ cells at 0 and 24 h after APAP administration as assessed by flow cytometric analysis. Data are expressed as the means ± SEM of three mice per group. *p < 0.05 vs. WT mice. (**C**) Representative flow cytometry plots for Ly6G^hi^/CD11b^hi^/ROS-positive cells in WT and BLT_1_^−/−^ mice at 0 and 24 h after APAP administration. (**D**) The number of Ly6G^hi^/CD11b^hi^/ROS-positive cells at 0 and 24 h after APAP administration as assessed by flow cytometric analysis. Data are expressed as the means ± SEM of three mice per group. *p < 0.05 vs. WT mice. (**E,F**) The mRNA levels of CD11b (**E**) and ICAM-1 (**F**) in the livers of WT and BLT_1_^−/−^ mice after APAP administration. Data are expressed as the means ± SEM of 5–6 mice per group. *p < 0.05 vs. WT mice.

Substantial APAP-induced liver injury in BLT_1_^−/−^ mice is correlated with enhanced expression of pro-inflammatory mediators and excessive accumulation of hepatic neutrophils. Neutrophil activation is essential for the aggravation of the injury. To examine the activation status of neutrophils recruited in the liver, we performed flow cytometric analysis. The number of Ly6G^hi^/CD11b^hi^ cells was higher (3.3-fold) in BLT_1_^−/−^ mice than in WT mice (Fig. 5A,B). Additionally, we examined the production of reactive oxygen species (ROS) in neutrophils (Fig. 5C). The number of ROS-producing Ly6G^hi^/CD11b^hi^ cells at 24 h was higher (3.0-fold increase) in BLT_1_^−/−^ mice than in WT mice (Fig. 5D). These results indicated that the accumulation of activated neutrophils into the liver was more extensive in BLT_1_^−/−^ mice than in WT mice.

Because neutrophil-mediated cytotoxicity is at least in part caused by the CD11b/intercellular adhesion molecule (ICAM)-1 pathway, we determined the hepatic mRNA expression of CD11b and ICAM-1. At 24 h after APAP administration, the mRNA levels of CD11b and ICAM-1 were higher in the livers of BLT_1_^−/−^ mice than in those of WT mice (Fig. 5E,F).

### Neutrophils from BLT_1_^−/−^ mice exacerbate APAP-induced liver injury in WT mice

**Figure 6:**
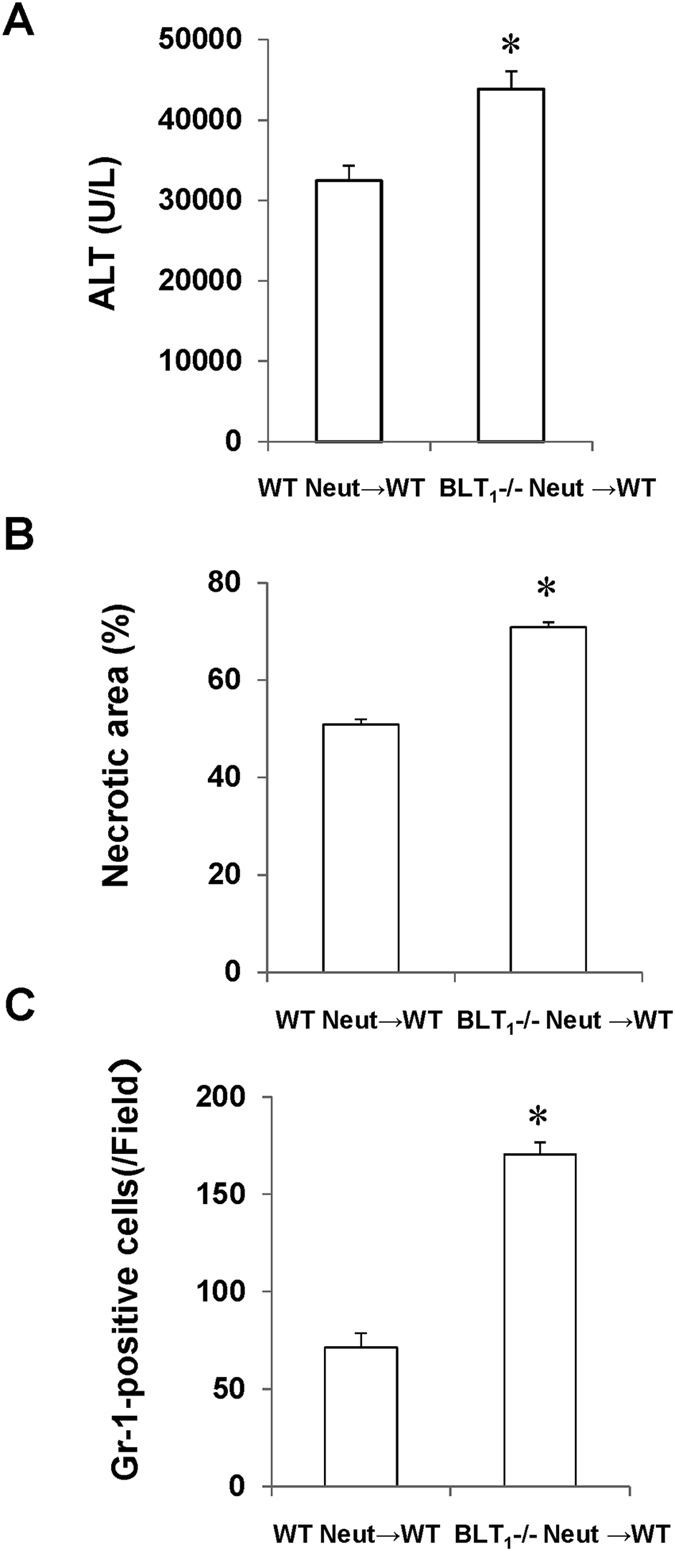
APAP-induced liver injury following the adoptive transfer of neutrophils from WT or BLT_1_^−/−^ mice. Murine neutrophils were isolated from the BM of WT or BLT_1_^−/−^ mice. BM neutrophils of each genotype were injected intravenously into WT or BLT_1_^−/−^ mice at 6 h after APAP treatment. The ALT level (**A**), hepatic necrotic area (**B**) and number of hepatic Gr-1-positive cells (**C**) in WT mice that received WT neutrophils (WT Neut → WT) or BLT_1_^−/−^ neutrophils (BLT_1_^−/−^ Neut → WT) were determined at 24 h after APAP administration. Data are expressed as the means ± SEM of five mice per group. *p < 0.05 vs. WT Neut → WT mice. Neut, neutrophils.

To further examine the contribution of BLT_1_ signalling in neutrophils to APAP-induced liver injury, we performed adoptive transfer experiments of purified BLT_1_-deficient neutrophils into WT mice treated with APAP. The isolated BLT_1_-deficient neutrophils from bone marrow (BM) were intravenously injected at 6 h after APAP administration^29^. Administration of BLT_1_-deficient neutrophils into WT mice significantly aggravated APAP-induced liver injury, as evidenced by increases in the ALT level and hepatic necrotic area in comparison with WT neutrophil-treated WT mice (Fig. 6A,B). The number of hepatic Gr-1-positive cells was higher in BLT_1_-deficient neutrophil-transferred WT mice than in WT neutrophil-transferred WT mice (Fig. 6C).

### Hepatic neutrophils from BLT_1_^−/−^ mice enhance the expression of MMP-9

**Figure 7:**
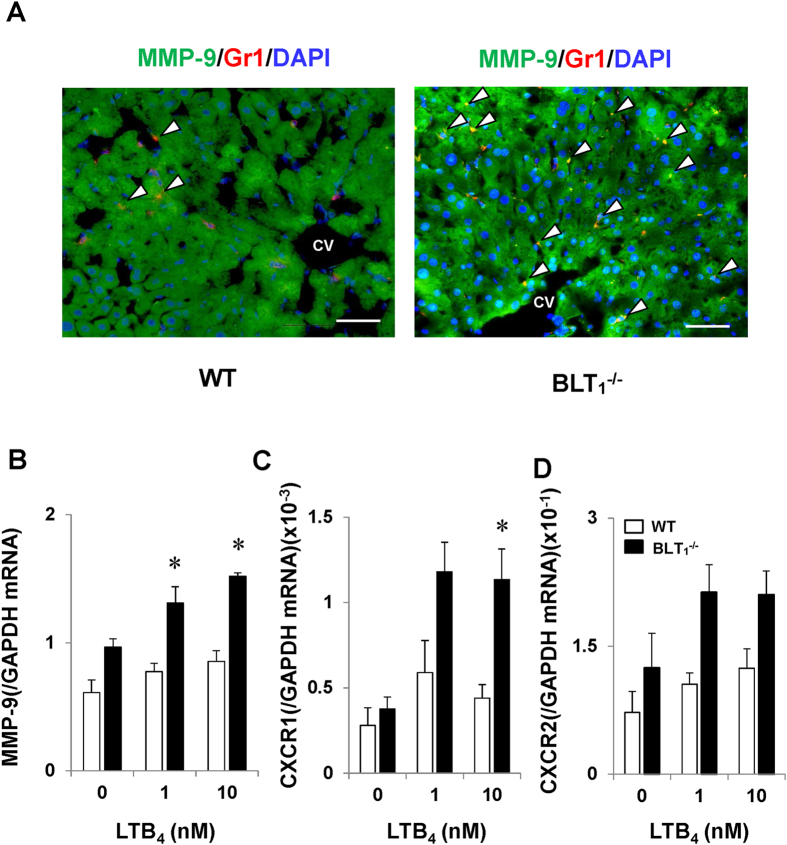
The expression of MMP-9 in neutrophils during APAP hepatotoxicity. (**A**) Double staining of liver sections with antibodies against MMP-9 (green) and Gr-1 (red) in WT and BLT1^−/−^ mice at 24 h after APAP administration. Arrow heads indicate double-labelled cells. CV, central vein. Bars = 50 μm. (**B–D**) The effects of LTB_4_ on the expression of MMP-9, CXCR1 and CXCR2 in BM-derived neutrophils from WT and BLT_1_^−/−^ mice. Isolated neutrophils were treated with LTB_4_, and the mRNA levels of MMP-9 (**B**), CXCR1 (**C**) and CXCR2 (**D**) were determined 1 h after incubation. Real-time quantitative RT-PCR assays were used to assess mRNA expression. Data are expressed as the means ± SEM of three independent experiments. *p < 0.05 vs. WT mice.

Recruited neutrophils in the livers of BLT_1_^−/−^ mice were correlated with APAP-induced liver injury. Because up-regulated MMP-9 can damage LSECs during APAP hepatotoxicity^30^,^31^, and MMP-9 released from neutrophils would be involved in sinusoidal injury during hepatitis^32^, we determined whether hepatic accumulated neutrophils during APAP hepatotoxicity enhanced the expression of MMP-9. Immunofluorescence analysis revealed that Gr-1-positive cells (neutrophils) in the liver were co-stained with MMP-9. The number of cells double-positive for Gr-1 and MMP-9 was higher in BLT_1_^−/−^ mice than in WT mice (Fig. 7A). We further examined whether neutrophils in BLT_1_^−/−^ mice up-regulate the expression of MMP-9 during APAP hepatotoxicity. To this end, isolated neutrophils from BM were stimulated with LTB_4_. Application of LTB_4_ enhanced MMP-9 mRNA expression in neutrophils from BLT_1_^−/−^ mice in comparison with WT mice (Fig. 7B). Treatment of isolated BLT1-deficient neutrophils with LTB_4_ also increased the mRNA level of CXCR1 (Fig. 7C), but not of CXCR2 (Fig. 7D). These results suggest that activated neutrophils in BLT_1_^−/−^ mice during APAP hepatotoxicity would damage LSECs through releasing ROS or the inflammatory mediator MMP-9.

### Effects of an anti-CXCL2 antibody on APAP-induced liver injury in BLT_1_^−/−^ mice

It is suggested that chemokines are responsible for the enhancement of hepatic neutrophil recruitment in BLT_1_^−/−^ mice. To further determine the role of chemokines in neutrophil recruitment and liver injury during APAP hepatotoxicity, BLT_1_^−/−^ mice were treated with a neutralising antibody against CXCL2. The number of Gr-1-positive cells was reduced by 35% in comparison with vehicle treatment (Fig. S4A). Concomitantly, the level of ALT in antibody-treated mice was decreased by 30% in comparison with vehicle-treated mice (Fig. S4B). We also examined the effect of the anti-CXCL2 antibody on pro-inflammatory mediators. RT-PCR analysis revealed that the hepatic levels of TNFα, IL-1β, IL-6 and MMP-9 in antibody-treated BLT_1_^−/−^ mice were reduced by 82%, 85%, 44% and 82%, respectively, in comparison with vehicle-treated BLT_1_^−/−^ mice (Fig. S4C–F).

### A minor role of BLT_2_ in APAP hepatotoxicity

The finding that BLT_1_^−/−^ mice displayed accelerated liver injury elicited by APAP suggests that signalling other than BLT_1_ signalling is involved in the injury. Because BLT_2_ is another receptor for LTB_4_, we treated BLT_1_^−/−^ mice with the BLT_2_-prone antagonist, LY255283, to examine whether BLT_2_ signalling is responsible for exacerbated APAP hepatotoxicity in BLT_1_^−/−^ mice. LY255283 failed to reduce the level of ALT in APAP-treated BLT_1_^−/−^ mice in comparison with vehicle administration (0.75% dimethyl sulfoxide (DMSO)) (Fig. S5). These results indicate that BLT_2_ signalling is unlikely to be involved in the enhancement of APAP-induced liver injury in BLT_1_^−/−^ mice.

## Discussion

In the present study, we demonstrated that genetic deletion or pharmacological inhibition of the BLT_1_ signalling pathway exacerbated APAP-induced liver injury. Additionally, BLT_1_^−/−^ mice were susceptible to APAP hepatotoxicity and displayed lower survival rates. Herein, we report the novel observation that BLT_1_ signalling unmasked potent preventive effects on liver injury elicited by APAP overdose. Specifically, the present findings reveal that, in contrast to WT mice, BLT_1_ deficiency promoted the increased recruitment of activated neutrophils into the livers of APAP-treated mice. In addition, BLT_1_-deficient neutrophils exhibited an enhanced ability to produce ROS and MMP-9 in comparison with WT neutrophils. These results indicate that BLT_1_ signalling protects the liver from APAP hepatotoxicity through inhibiting the excessive accumulation of neutrophils into the liver.

BLT_1_ signalling exerts pro-inflammatory actions through the enhancement of neutrophil recruitment in several inflammatory diseases^19^,^26^,^27^,^28^. Indeed, BLT_1_^−/−^ mice are protected from the development of rheumatoid arthritis^26^, spinal cord injury^27^ and atopic dermatitis^28^, which are associated with decreases in the accumulation of neutrophils into inflammatory sites and in the production of chemokines and cytokines. These studies also indicate that BLT_1_ signalling in the infiltrated neutrophils is essential for the development of these inflammatory diseases. In liver disease models, BLT_1_ signalling is responsible for liver microcirculatory dysfunction including leukocyte adhesion during endotoxemia^33^. During warm hepatic ischemia/reperfusion (I/R), BLT_1_^−/−^ mice exhibit less accumulation of neutrophils in the liver; however, there is no significant difference in the degree of hepatic I/R injury between the phenotypes^34^. Collectively, it is suggested that BLT_1_ signalling mediates neutrophil infiltration at local inflammatory sites to enhance inflammation and injury. BLT_1_ signalling has potent pro-inflammatory effects in inflammatory diseases^19^. By contrast, the current study demonstrated that BLT_1_ signalling suppressed the excessive infiltration of neutrophils into inflammatory sites to protect the liver from APAP hepatotoxicity. BLT_1_ signalling in neutrophils has a protective action against the progression of APAP overdose-induced liver injury. Thus, BLT_1_ signalling exerts anti-inflammatory actions under certain pathological conditions such as APAP hepatotoxicity, while pro-inflammatory effects of BLT_1_ in other inflammatory disease models have been established.

APAP hepatotoxicity triggers an extensive inflammatory response with cytokine formation^4^,^7^. The inflammatory response to APAP depends on the injury insult. Our data demonstrated that enhanced APAP-induced liver injury in BLT_1_^−/−^ mice was associated with up-regulated expression of pro-inflammatory cytokines including TNFα, IL-1β and IL-6 (Fig. 4A–C). Aggravated liver injury is also correlated with increases in the accumulation of neutrophils into the injured liver. The extensive hepatic centrilobular necrosis during APAP hepatotoxicity results in extensive release of damage-associated molecular patterns (DAMPs) from cells undergoing necrosis^4^,^35^,^36^. DAMPs trigger the generation of pro-inflammatory cytokines from macrophages, which leads to the recruitment of neutrophils into the injured liver^4^. As a result, APAP hepatotoxicity causes inflammatory responses including generation of pro-inflammatory mediators^7^,^11^. Enhanced liver injury through generation of pro-inflammatory mediators would cause further neutrophil accumulation into the injured liver. Therefore, attenuated hepatic necrosis during APAP toxicity will reduce DAMP release and consequently reduce cytokine formation and neutrophil infiltration. The levels of pro-inflammatory mediators reflect the degree of neutrophil activation and accumulation in the liver. However, it remains to be elucidated whether neutrophil recruitment is a cause or consequence of the exacerbation of APAP-induced liver injury^4^,^6^,^8^,^9^,^29^.

The role of neutrophils in the evolution of APAP-induced liver injury is controversial. Depletion of neutrophils by treatment with an anti-Gr-1 antibody and pharmacological blockage of neutrophil chemotactic receptor (CXCR2) protect mice against APAP-induced liver injury^8^. These findings suggest that neutrophils have deleterious functions in the innate immune system during APAP hepatotoxicity. By contrast, numerous interventions that prevent neutrophil cytotoxicity, such as blocking antibodies against CD18, gene deficiency of CD18 and ICAM-1, inhibitors of NADPH oxidase, and deficiency of NADPH oxidase, are ineffective in attenuating APAP hepatotoxicity^4^,^9^. This indicates that accumulation of neutrophils is not responsible for liver injury elicited by APAP. The results of the present study showed that BLT_1_ signalling protects the liver from APAP toxicity through inhibiting the excessive accumulation of activated neutrophils into the liver, suggesting that BLT_1_ signalling deficiency in neutrophils would contribute to the development of APAP hepatotoxicity.

According to the results by Liu *et al*.^6^, depletion of neutrophils by treatment with an anti-Gr-1 antibody protects the liver from APAP (500 mg/kg) toxicity, which is a higher dose of APAP used in the present study. This suggests that accumulated hepatic neutrophils are essential for the development and severity of liver injury elicited by even a high dose of APAP (500 mg/kg). Because the severity of liver injury elicited by APAP is dose-dependent, the neutrophil response to APAP also appears to be in a dose-dependent manner. In addition, the survival rate of BLT_1_^−/−^ mice treated with APAP (300 mg/kg) was 48.6% by 72 h (Fig. 1A), while that of WT mice (C57Bl/6 mice) treated with APAP (500 mg/kg) was 58% by 72 h^6^. Based on these observations, it may be conceivable that the mortality of BLT_1_^−/−^ mice treated with APAP (500 mg/kg) would be quite severe, and it would be hard to evaluate the role of neutrophils in BLT_1_^−/−^ mice treated with higher doses of APAP. Therefore, we selected the moderate dose of APAP (300 mg/kg) in the current study.

The priming or activation of neutrophils, as evidenced by reactive oxygen formation, is required for neutrophil-mediated liver injury^4^. Activated neutrophils in inflammation exert their cytotoxic effects through the generation of ROS. However, it is indicated that hepatic neutrophils are not activated in terms of ROS production during APAP hepatotoxicity^4^,^9^, suggesting that neutrophils recruited into the injured liver play a minor role in the aggravation of liver injury elicited by APAP. By contrast, the present study revealed that BLT_1_-deficient neutrophils (Ly6G^hi^/CD11b^hi^) recruited into the injured liver at 24 h after APAP treatment displayed higher levels of ROS production than WT neutrophils (Fig. 5C,D), indicating that neutrophils from BLT_1_^−/−^ mice are activated at 24 h post-APAP administration. Activated BLT_1_-deficient neutrophils in the liver were associated with enhanced liver injury elicited by APAP, indicating that BLT_1_ signalling contributes to prevention of APAP hepatotoxicity through the inactivation of neutrophils recruited into the injured liver. Of interest, adoptive transfer of BLT_1_-deficient neutrophils into WT mice treated with APAP exacerbated liver injury (Fig. 6A,B), indicating that BLT_1_ signalling in neutrophils has a protective action against liver injury elicited by APAP overdose. The current study also revealed that extensive accumulation of neutrophils in the livers of BLT_1_^−/−^ mice treated with APAP was associated with up-regulated hepatic expression of CD11b and ICAM-1 (Fig. 5E,F). ICAM-1 is up-regulated in LSECs and is induced in hepatocytes in areas of parenchymal injury, where ICAM-1 induction correlates with the degree of liver damage^37^. Up-regulation of ICAM-1 is essential for neutrophil extravasation into the extra-sinusoidal space to attack hepatocytes^38^. However, a deficiency in CD18 on the surface of neutrophils or in ICAM-1 has no impact on APAP-induced liver injury in mice^4^.

Taken together, hepatic neutrophil recruitment is a characteristic feature of immune responses in APAP hepatotoxicity; however, diverging results concerning the role of neutrophils in APAP-induced liver injury need to be resolved. Investigating the association between neutrophils and other cell types of the immune system, such as resident or infiltrating macrophages and monocytes, might provide new insights into the contribution to innate immunity during APAP hepatotoxicity^12^.

Haemorrhagic hepatic necrosis is a characteristic of liver injury elicited by APAP overdose^39^. In BLT_1_^−/−^ mice and ONO-0457-treated WT mice, recruitment of neutrophils was correlated with haemorrhagic necrosis, which could lead to animal death and a poor animal survival rate due to severe impairment in hepatic microcirculation^11^,^39^. Thus, it is suggested that BLT_1_ signalling is critical for suppressing inflammatory responses including excessive accumulation of neutrophils and for protecting LSECs from injury in response to APAP. During APAP hepatotoxicity, LSECs are injured as evidenced by gap formation in their cytoplasm. The formation of gaps results in the penetration of erythrocytes, severe haemorrhage and liver microcirculatory dysfunction^11^,^39^. Although the mechanisms by which administration of APAP damages LSECs are not entirely clear^31^, we found that the production of ROS and MMP-9 by recruited neutrophils in BLT_1_^−/−^ mice was enhanced in comparison with those in WT mice^40^. Isolated neutrophils from BLT_1_^−/−^ mice expressed a higher level of MMP-9 in response to LTB_4_ in cell culture (Fig. 7B). Excessive ROS and MMPs could be responsible for injury to sinusoids through proteolytic cleavage of the LSEC membrane^30^,^31^,^32^. It is also suggested that MMP-9 facilitates the migration of leukocytes into inflamed livers^41^,^42^. Disruption of BLT_1_ signalling induces more severe APAP hepatotoxicity with excessive activated neutrophil recruitment, probably due to the impairment of neutrophil function maintained by BLT_1_ receptors. However, the mechanisms by which BLT_1_ signalling regulates the release of ROS and MMP-9 remain to be elucidated. We and others previously reported that VEGFR1 signalling is essential for maintaining the integrity of LSECs in terms of their function and structure^11^,^43^,^44^. The current study also found that VEGFR1 expression was reduced in the livers of BLT_1_^−/−^ mice during APAP hepatotoxicity, suggesting that hepatic haemorrhage partly results from down-regulated expression of VEGFR1 in the livers of BLT_1_^−/−^ mice.

The present study demonstrates that BLT_1_ deficiency and a BLT_1_ antagonist exacerbated APAP-induced liver injury, as indicated by increases in the ALT level, hepatic centrilobular necrotic area, haemorrhagic area and expression of pro-inflammatory mediators (Fig. 3). BLT_1_^−/−^ mice were susceptible to APAP hepatotoxicity and displayed lower survival rates (Fig. 1A). Thus, BLT_1_ signalling negatively regulates severe liver injury elicited by APAP overdose. The current study also indicates that hepatic neutrophil activation and recruitment are essential for the development of severe liver injury in BLT_1_^−/−^ mice after APAP administration. A key issue that remained to be addressed is how BLT_1_ deficiency promotes increased neutrophil infiltration into the liver at 24 h after APAP treatment. To address this question, we determined the levels of chemokines (CXCL1 and CXCL2) and their receptors (CXCR1 and CXCR2) because chemokine gradients of CXCL1 and CXCL2 guide neutrophils through CXCR1 and CXCR2 into necrotic areas^45^. The hepatic CXCL2 level was significantly higher in BLT_1_^−/−^ mice than in WT mice at 24 h post-APAP treatment (Fig. 2E). In addition, treatment of BLT_1_^−/−^ mice with an anti-CXCL2 antibody attenuated inflammatory responses to APAP, including reductions in the levels of ALT, neutrophil recruitment and inflammatory mediators (Fig. S4). Although we have not examined the sources of CXCL2 during APAP hepatotoxicity, hepatocytes and Kupffer cells can generate CXC chemokines^45^,^46^. In addition, the mechanisms by which the absence of BLT_1_ signalling enhances CXCL2 in the liver after APAP administration are not clear.

A lack of BLT_1_ signalling amplified APAP-induced liver injury, suggesting that signalling other than BLT_1_ signalling is involved in the enhanced liver injury. Because BLT_2_, as well as BLT_1_, is a receptor for LTB_4_, we examined whether BLT_2_ contributes to the exacerbated APAP hepatotoxicity in BLT_1_^−/−^ mice. However, the BLT_2_-prone antagonist failed to affect the exacerbated APAP hepatotoxicity in BLT_1_^−/−^ mice (Fig. S5). Although BLT_1_ signalling appears to be essential for protection against APAP hepatotoxicity, the mediator of exacerbated APAP hepatotoxicity in BLT_1_^−/−^ mice remains to be clarified.

A recent report^47^ showed that a lack of the 5-LOX gene and pharmacological inhibition of 5-LOX synthase reduce APAP-induced liver injury, as demonstrated by decreases in the ALT level and hepatic necrosis. The protective effect of 5-LOX inhibition on APAP hepatotoxicity is attributed to attenuation of APAP bioactivation and oxidative stress. In their study, the effect of 5-LOX on neutrophil recruitment in response to APAP was not addressed. Because LTB_4_ is synthetised through 5-LOX, our results appear to contradict their results. However, 5-LOX is high upstream in the LTB_4_/BLT_1_ signalling pathway. 5-LOX produces not only LTB_4_, but also other LTs such as LTC_4_, LTD_4_ and LTE_4_, and these LTs act through different receptors. Additionally, 5-LOX metabolises arachidonic acid to 5-hydroperoxyeicosatetraenoic acid, which is the precursor for LTs and a bioactive product. Thus, it is conceivable that the effects of selective inhibition of a 5-LOX-derived lipid mediator receptor on APAP-induced liver injury are different from those of the broad inhibition of 5-LOX-derived lipid mediators. Although BLT_1_ inhibition is a selective approach, inhibition of BLT_1_ signalling has adverse effects and is not suitable for use as a therapeutic tool to treat APAP hepatotoxicity.

In conclusion, we clarified the role of BLT_1_ signalling in APAP hepatotoxicity in mice. BLT_1_ signalling dampens the progression of APAP-induced liver injury through inhibiting an excessive accumulation of activated neutrophils. The present study suggests that the development of a specific agonist for BLT_1_ signalling in neutrophils could be useful for the prevention of APAP hepatotoxicity.

## Methods

### Animals

Male C57Bl/6 WT mice (8 weeks old) were obtained from Crea Japan (Tokyo, Japan). Male BLT_1_-knockout (BLT_1_^−/−^) mice (8 weeks old) were developed previously^48^. Mice were maintained at constant humidity (60 ± 5%) and temperature (25 ± 1 °C) on a 12 h light/dark cycle. All animals were provided food and water *ad libitum*. All experimental procedures were approved by the Animal Experimentation and Ethics Committee of the Kitasato University School of Medicine, and were performed in accordance with the guidelines for animal experiments set down by the Kitasato University School of Medicine.

### Animal procedures

Animals were fasted overnight and then intraperitoneally received 300 mg/kg APAP (Sigma-Aldrich, St. Louis, MO, USA) dissolved in warm saline (20 mg/mL). Some animals received a single i.p. injection of 10 mg/kg of the selective BLT_1_ antagonist ONO-4057 (Ono Pharmaceutical, Osaka, Japan) or vehicle (phosphate-buffered saline (PBS)) concomitantly with APAP administration^27^,^49^. Other animals were treated with LY255283 (5 mg/kg, i.p.), the BLT_2_-prone antagonist (Cayman, Ann Arbor, MI, USA)^19^, dissolved in 0.75% DMSO prepared in saline or the vehicle alone (10 mL/kg) 1 h before treatment with APAP^50^. For some experiments, BLT_1_^−/−^ mice were given a single intravenous (i.v.) injection of an anti-mouse CXCL2 antibody (5 mg/kg) (R&D systems, Minneapolis, MN, USA) or vehicle (control IgG) concomitantly with APAP administration^51^.

### Experimental protocols

At the indicated time points, animals were anesthetised with pentobarbital sodium (50 mg/kg, i.p.). Blood was drawn from the heart and then centrifuged. Serum ALT activity was measured in a Dri-Chem 4000 Chemistry Analyser System (Fujifilm, Tokyo, Japan). Immediately after blood collection, the livers were excised and rinsed in saline. A small section of each liver was placed in 4% paraformaldehyde, and the remaining liver was frozen in liquid nitrogen and stored at −80 °C.

### Histology and immunohistochemistry

Excised liver tissues were fixed immediately with 4% paraformaldehyde prepared in 0.1 M sodium phosphate buffer (pH 7.4) for histological analysis^34^,^44^. Sections (4 μm thick) were prepared from paraffin-embedded tissue and subjected to either haematoxylin and eosin (H&E) staining or immunostaining. The level of necrosis (as a percentage of the total area) was estimated by measuring the necrotic area relative to the entire histological section, and the necrotic area was analysed with a VH analyser (KEYENCE, Osaka, Japan). The haemorrhagic area was also determined to quantify the extent of haemorrhage. The results were expressed as a percentage.

### Immunofluorescence staining

Tissue samples were fixed with periodate-lysine-paraformaldehyde fixative at room temperature for 3 h. Following cryoprotection with 30% sucrose prepared in 0.1 M phosphate buffer (pH 7.2), sections (approximately 10–20 μm thick) were cut in a cryostat. Sections were then incubated with 1% bovine serum albumin (BSA) prepared in PBS at room temperature for 1 h to block non-specific binding, followed by incubation with a rabbit anti-mouse 5-LOX polyclonal antibody (Novus Biologicals Inc., Littleton, CO, USA), a rat anti-mouse Gr-1 IgG2b monoclonal antibody (mAb) (AbD Serotec, Raleigh, NC, USA), a rat anti-mouse CD68 IgG2a mAb (AbD Serotec), an anti-CD31 rabbit polyclonal antibody (Abcam, Cambridge, MA, USA) and a goat anti-mouse serum albumin polyclonal IgG antibody (Abcam). After washing three times in PBS, the sections were incubated with a mixture of the following secondary antibodies for 1 h at room temperature: Alexa Fluor^®^ 488-conjugated donkey anti-rat IgG (1:500; Molecular Probes, Eugene, OR, USA) and Alexa Fluor^®^ 594-conjugated donkey anti-rabbit IgG (1:500; Molecular Probes). As a negative control, sections were incubated in 1% BSA prepared in PBS in the absence of a primary antibody. Images were captured with a fluorescence microscope (Biozero BZ-9000 Series; KEYENCE). After labelling, six low-power optical fields (200× magnification) were randomly selected and the number of positive cells was counted. At least five animals were analysed per marker.

### Real-time RT-PCR analysis

Transcripts encoding 5-LOX, BLT_1_, BLT_2_, IL-6, TNFα, IL-1β, MMP-2, MMP-9, PAI-1, CXCL1, CXCL2, CXCR1, CXCR2 and glyceraldehyde-3-phosphate dehydrogenase (GAPDH) were measured by real-time RT-PCR. Briefly, total RNA was extracted from liver tissues and homogenised in TRIzol reagent (Invitrogen, Carlsbad, CA, USA). RNA expression was measured in a BioPhotometer (Eppendorf Co. Ltd., Tokyo, Japan). The primers used for real-time PCR were designed using Primer 3 software (http://primer3.sourceforge.net/) based on data from GenBank, and the sequences are listed in Supplementary Table 1. Data were normalised to the expression level of GAPDH in each sample.

### GSH/GSSG assay

Organs were removed and snap-frozen in liquid nitrogen. Frozen tissues were homogenised in Tissue Extraction Reagent I (Invitrogen) and 0.05% protease inhibitor cocktail (Sigma-Aldrich), and centrifuged to separate the supernatant. Hepatic GSH and GSSG were measured colorimetrically with a BIOXYTECH GSH/GSSG Assay kit (OxisResearch, Portland, OR, USA).

### Isolation of intrahepatic leukocytes

Under anaesthesia with pentobarbital sodium solution (50 mg/kg, i.p.), the liver was perfused through the portal vein with perfusion buffer (10 mL, 1 × Hank’s balanced salt solution). The excised livers were immediately placed in ice-cold RPMI, minced with scissors into small pieces and incubated in RPMI containing 0.05% collagenase (Type IV; Sigma Chemical Co., St. Louis, MO, USA) at 37 °C for 20 min. The tissue was then pressed through a 70 μm cell strainer. Hepatic leukocytes were isolated from liver homogenates by density-gradient centrifugation with 33% percoll™ (GE Healthcare Life Sciences, Piscataway, NJ, USA) as previously reported^52^. Viable, nucleated cells were counted by trypan blue exclusion and brought to a uniform cell density.

### Flow cytometric analysis

Cells were incubated with the 2.4G2 mAb (anti-cγRIII/II) to block non-specific binding of the primary mAb. Then, saturating concentrations of PE-labelled-anti-Ly6G (BioLegend, San Diego, CA, USA) and PE-Cy7-labelled-CD11b (BioLegend) antibodies were added. Tubes were placed in the dark on ice for 30 min. Pellets were washed twice with PBS. Samples were measured on FACSVerse™ (BD, Franklin Lakes, NJ, USA). Ly6G^hi^/CD11b^hi^ cells, which were identified as neutrophils, were gated. The data were analysed using Kaluza software v1.3 (Beckman Coulter, South Kraemer Boulevard Brea, CA, USA).

### Assay of reactive oxygen production

Hydrogen peroxide production by isolated leukocytes was examined by assessing oxidation of dihydrorhodamine to fluorescent rhodamine. Cells were incubated with diphenyleneiodonium (10 μL) for 20 min. Phorbol 12-myristate 13 acetate (2 μM) was added, and cells were incubated for 10 min at 37 °C. Dihydrorhodamine-123 (4 μM) was added, and cells were incubated for 5 min at 37 °C. After blocking, cells were stained with saturating concentrations of PE-labelled-anti-Ly6G and PE-Cy7-labelled-CD11b antibodies. Samples were measured on FACSVerse™, and data were analysed using Kaluza software v1.3.

### Adoptive transfer of BM-derived neutrophils

Mouse BM cells were collected by flushing the femur and tibia of WT and BLT_1_^−/−^ mice with PBS. Neutrophils were isolated from BM cells using the Neutrophil Isolation Kit (Miltenyi Biotec, Auburn, CA, USA). Purified BM-derived neutrophils were adoptively transferred through an i.v. injection of 4 × 10^6^ cells per mouse as described previously^29^ at 6 h after APAP treatment.

### Cell culture

Isolated neutrophils from BM (2 × 10^6^ cells) were stimulated with LTB_4_ (Cayman Chemical, Ann Arbor, MI, USA) for 1 h at 37 °C. Collected cells were immediately immersed in TRIzol reagent (Invitrogen) and homogenised. qPCR amplification was performed as described above.

### Statistical analysis

All results are expressed as the mean ± standard error of the mean (SEM). All statistical analyses were performed using GraphPad Prism software, version 6.01 (GraphPad Software, La Jolla, CA, USA). The Student’s t-test was used to compare data between two groups, and a one-way analysis of variance followed by Bonferroni’s post-hoc test was used to compare data between multiple groups. The survival rates of WT and BLT_1_^−/−^ mice were compared by the Kaplan-Meier survival and log-rank tests. A p-value < 0.05 was considered statistically significant.

## Additional Information

**How to cite this article**: Kojo, K. *et al*. BLT_1_ signalling protects the liver against acetaminophen hepatotoxicity by preventing excessive accumulation of hepatic neutrophils. *Sci. Rep.* **6**, 29650; doi: 10.1038/srep29650 (2016).

## Supplementary Material

Supplementary Information
