# Supplementary material for: BLT1 signalling protects the liver against acetaminophen hepatotoxicity by preventing excessive accumulation of hepatic neutrophils
Source: Sci Rep. 2016 Jul 11;6:29650. doi: 10.1038/srep29650 (PMC4939602; doi:10.1038/srep29650)

Supplementary information

BLT_1_ signalling protects the liver against acetaminophen hepatotoxicity by preventing excessive accumulation of hepatic neutrophils

Ken Kojo^1,2^, Yoshiya Ito^2^, Koji Eshima^3^, Nobuyuki Nishizawa^1,2^, Hirotoki Ohkubo^4^, Takehiko Yokomizo^5^, Takao Shimizu^6^, Masahiko Watanabe^2^, Masataka Majima^1,*^

Departments of Pharmacology^1^, Surgery^2^, Immunology^3^ and Cardiovascular Surgery^4^, Kitasato University School of Medicine, Kanagawa 252-0374, Japan

Department of Biochemistry^5^, Juntendo University School of Medicine, Tokyo 113-8421, Japan

Department of Lipid Signaling^6^, National Center for Global Health and Medicine,

Tokyo 162-0052, Japan

Supplementary Table S1. The primers used for reverse transcription and quantitative PCR reactions.

| Gene | Forward primer sequence (5'–3') | Reverse primer sequence (5'–3') |
| --- | --- | --- |
| 5LOX | TCATTGAGAAGCCAGTGAAGG | GTTGGGAATCCTGTCTGGTGA |
| BLT1 | GGCTGCAAACACTACATCTCC | TCAGGATGCTCCACACTACAA |
| BLT2 | AGCCTTGGCTTTCTTCAGTTC | CCCTCGAAGAGTCGAGTAAGG |
| CXCL1 | AAACCGAAGTCATAGCCACAC | GGGGACACCTTTTAGCATCTT |
| CXCL2 | ATCCAGAGCTTGAGTGTGACG | GCCTTGCCTTTGTTCAGTATC |
| CXCR1 | GGCATCTGGGGTCTATCTTTG | TCATCCGAAAATCTGTTGTGG |
| CXCR2 | AACAATACATCCCGTTTGAGG | AGTGTGAACCCGTAGCAGAAC |
| CD11b | TCCTGCGCCTCAATTATACAC | CTGAGCATCCATAGCCAGAAC |
| ICAM-1 | TCGATCTTCCAGCTACCATCC | TCCAGGGAGCAAAACAACTTC |
| TNFα | TCTTCTCATTCCTGCTTGTGG | GATCTGAGTGTGAGGGTCTGG |
| IL1β | TACATCAGCACCTCACAAGCA | CCAGCCCATACTTTAGGAAGA |
| IL-6 | CAAAGCCAGAGTCCTTCAGAG | TAGGAGAGCATTGGAAATTGG |
| CYP2E1 | ATGGGGAAACAGGGTAATGAG | CAGAAAGGTAGGGTCAAAAGG |
| VEGFR1 | GATGAAGTTCCCCTGGATGAG | TTGAACGACTTTCCCAAAAGC |
| VEGF-A | ACGACAGAAGGAGAGCAGAAG | ATGTCCACCAGGGTCTCAATC |
| MMP-2 | TTCAAGGACCGGTTTATTTGG | CACAGCGTCAATCTTTTCTGG |
| MMP-9 | CCCATGTCACTTTCCCTTCAC | GCCGTCCTTATCGTAGTCAGC |
| PAI-1 | CTCCACAGCCTTTGTCATCTC | GATTGTCTCTGTCGGGTTGTG |
| GAPDH | ACATCAAGAAGGTGGTGAAGC | AAGGTGGAAGAGTGGGAGTTG |

**Supplementary Figure legends**

Supplementary Figure 1. Hepatic neutrophil accumulation in mice at 24 h after APAP treatment.

(A) Representative immunofluorescence staining for Gr-1 (green) in WT livers (A) at 24 h after APAP administration. Nuclei are stained with DAPI (blue). The area surrounded by the white dotted line indicates the centrilobular necrotic area. CV, central vein. Scale bar = 50 μm. (B) Representative immunofluorescence staining for Gr-1 (green) in BLT_1_^-/-^ livers at 24 h after APAP administration. Nuclei are stained with DAPI (blue). Livers from BLT_1_^-/-^ mice showed neutrophils extravasated (arrow heads) into the parenchymal tissue as well as neutrophils remained in the sinusoids (arrows). Nuclei are stained with DAPI (blue). CV, central vein. Scale bar = 50 μm.

Supplementary Figure 2. Hepatic expression of BLT_1_ and 5-LOX in WT and BLT_1_^-/-^ mice during APAP hepatotoxicity.

(A) Expression of BLT_1_ mRNA in livers of WT and BLT_1_^-/-^ mice after APAP administration. Data are expressed as the means ± SEM of six mice per group. (B) Hepatic expression of BLT_2_ mRNA in WT and BLT_1_^-/-^ mice after APAP administration. Data are expressed as the means ± SEM of six mice per group. (C) Hepatic expression of 5-LOX mRNA in WT and BLT_1_^-/-^ mice after APAP administration. Data are expressed as the means ± SEM of six mice per group. (D) Representative immunofluorescence staining for 5-LOX (green) in WT livers at 24 h after APAP administration. CV, central vein; PV, portal vein. Scale bar = 50 μm. (E–H) Double staining of liver sections from WT mice with antibodies against 5-LOX (green) and CD31 (red) (E), albumin (red) (F), CD68 (red) (G) or Gr-1 (red) (H) at 24 h after APAP administration. Arrow heads indicate double-labelled cells. Bars = 50 μm.

Supplementary Figure 3. Hepatic CYP2E1 mRNA expression and GSH content in WT and BLT_1_^-/-^ mice during APAP hepatotoxicity.

(A) Hepatic CYP2E1 mRNA expression was measured by real-time RT-PCR. Data are expressed as the means ± SEM of six mice per group. (B–D) Hepatic GSH (B) and GSSG (C) contents were measured, and the ratio of GSSG to GSH (D) was calculated. Data are expressed as means ± SEM of 4–6 mice per group.

Supplementary Figure 4. Treatment of BLT_1_^-/-^ mice with an anti-CXCL2 neutralising antibody attenuates APAP-induced hepatic inflammatory responses.

(A, B) Effects of treatment with an anti-CXCL2 neutralising antibody on the recruitment of neutrophils (A) and the ALT level (B) at 24 h after APAP administration. Data are expressed as the means ± SEM of 5–6 mice per group. *: p < 0.05 vs. control IgG (Veh)-treated WT mice. Veh, vehicle. (C–F) Effects of treatment with an anti-CXCL2 neutralising antibody on pro-inflammatory mediators including TNFα (C), IL-1β (D), IL-6 (E) and MMP-9 (F) at 24 h after APAP administration. The mRNA levels in livers of BLT1^-/-^ mice treated with an anti-CXCL2 neutralising antibody and control IgG were determined by real-time PCR. Data are expressed as the means ± SEM of 5–6 mice per group. *: p < 0.05 vs. control IgG (Veh)-treated WT mice. Veh, vehicle.

Supplementary Figure 5. Effects of treatment with a BLT_2_-prone antagonist, LY255283, on APAP-induced liver injury in BLT_1_^-/-^ mice.

BLT_1_^-/-^ mice were treated with LY255283 dissolved in 0.75% DMSO prepared in saline or the vehicle alone (10 mL/kg) 1 h before APAP administration. Serum ALT levels were determined at 24 h after APAP administration. Data are expressed as the means ± SEM of 5–6 mice per group.


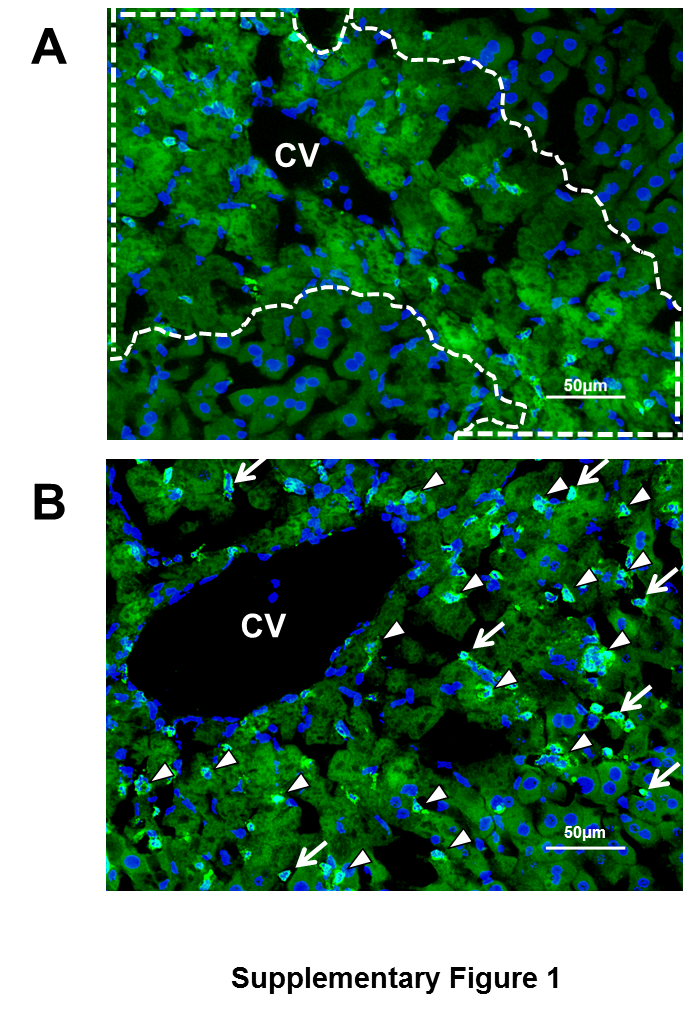


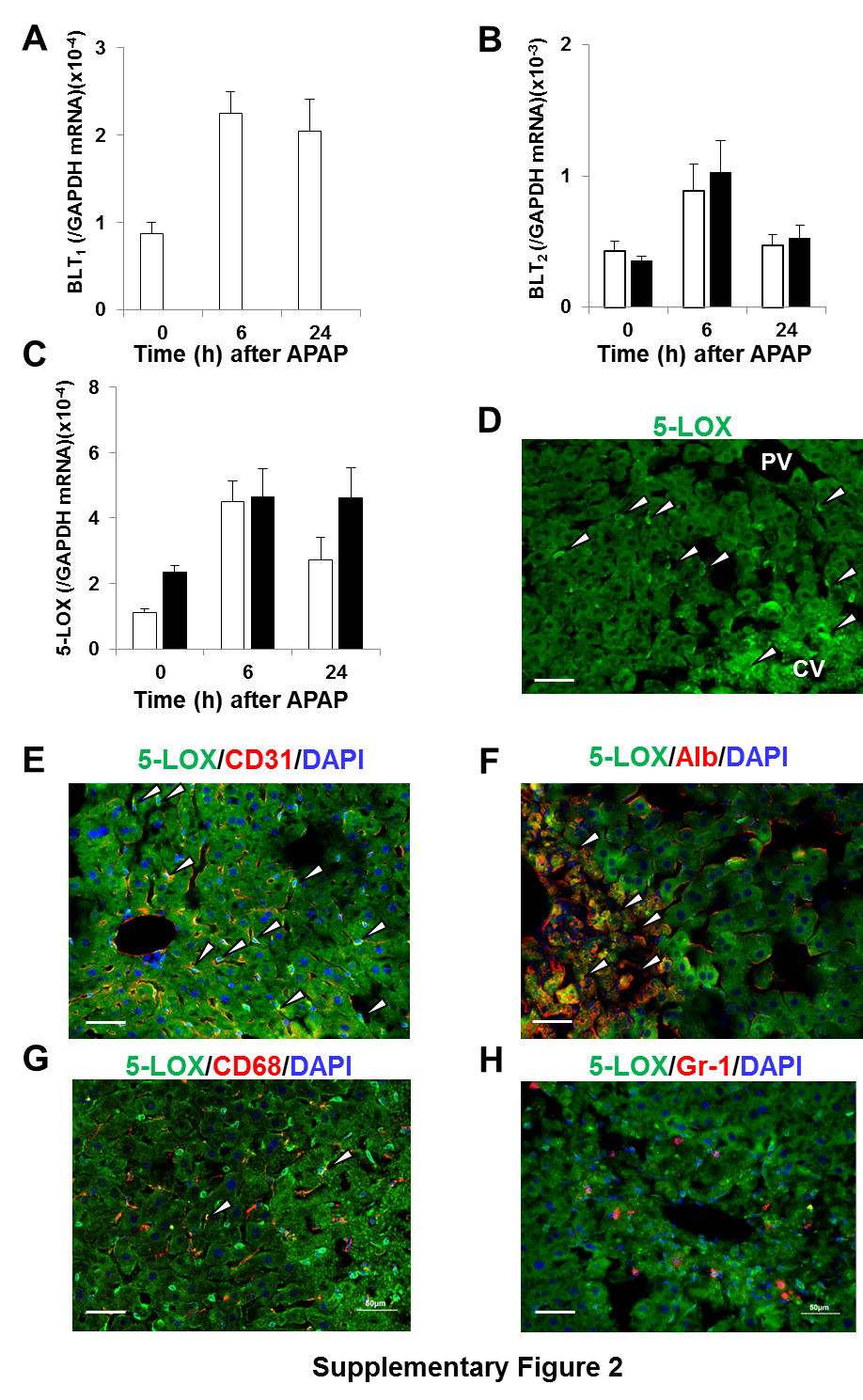


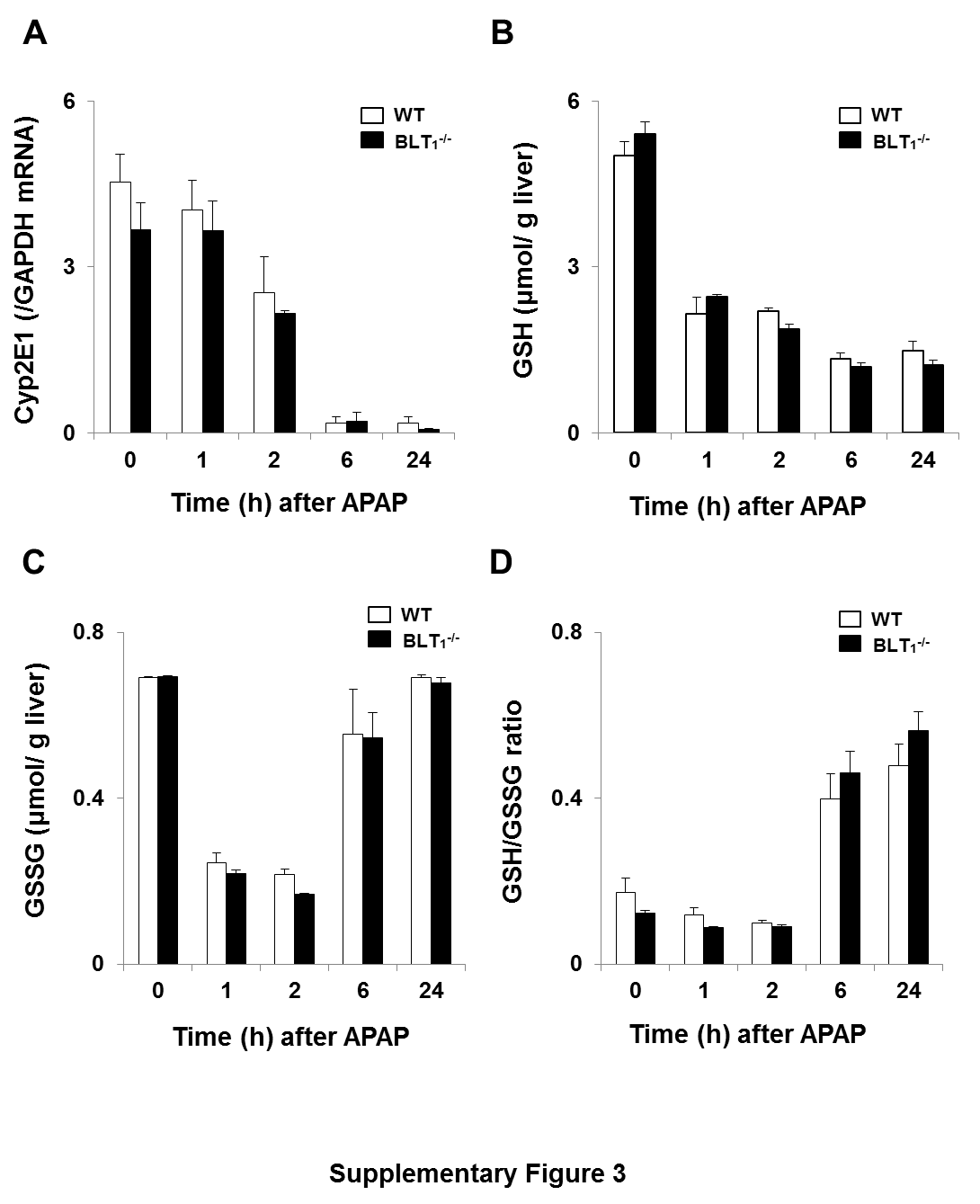


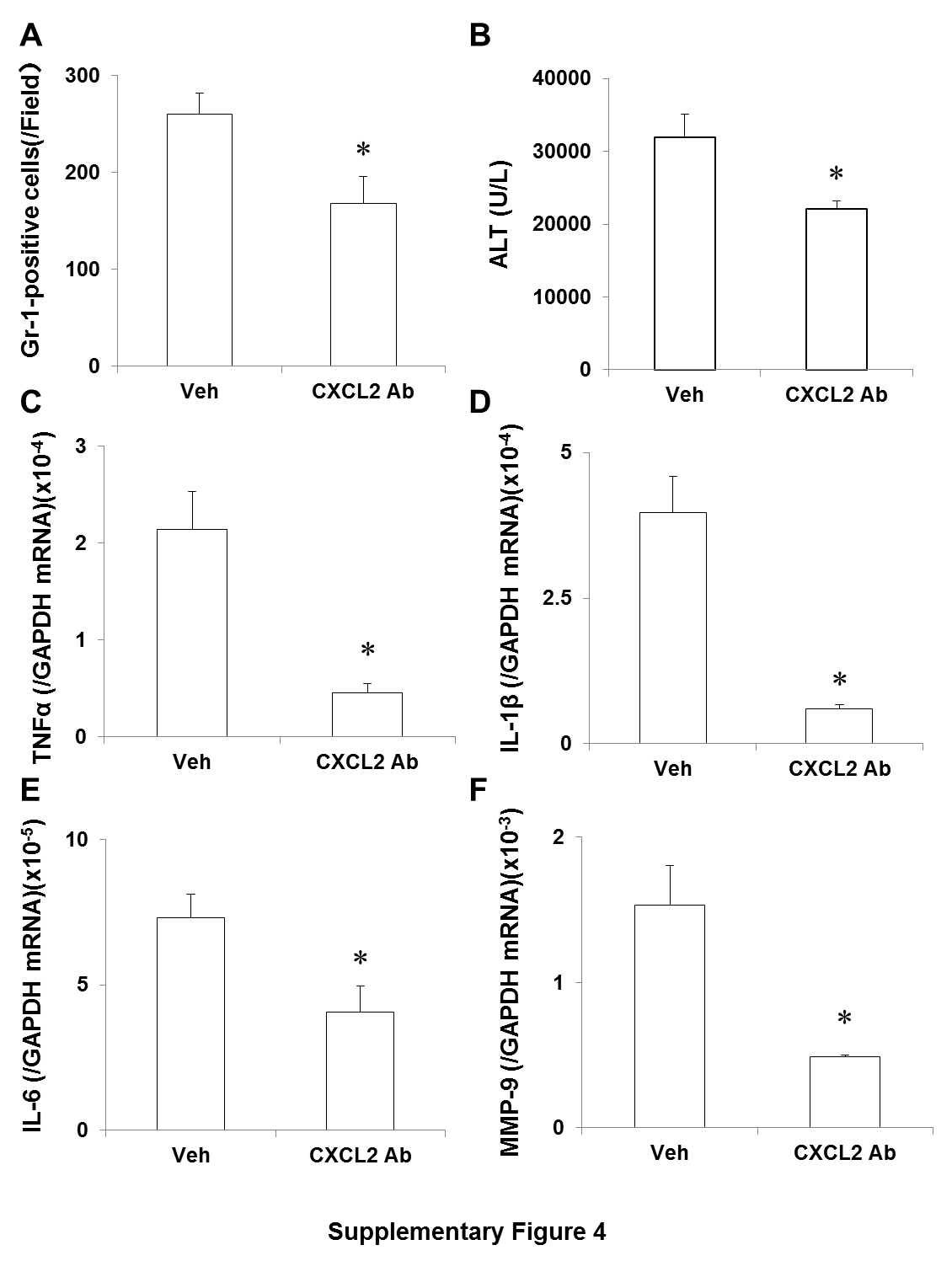


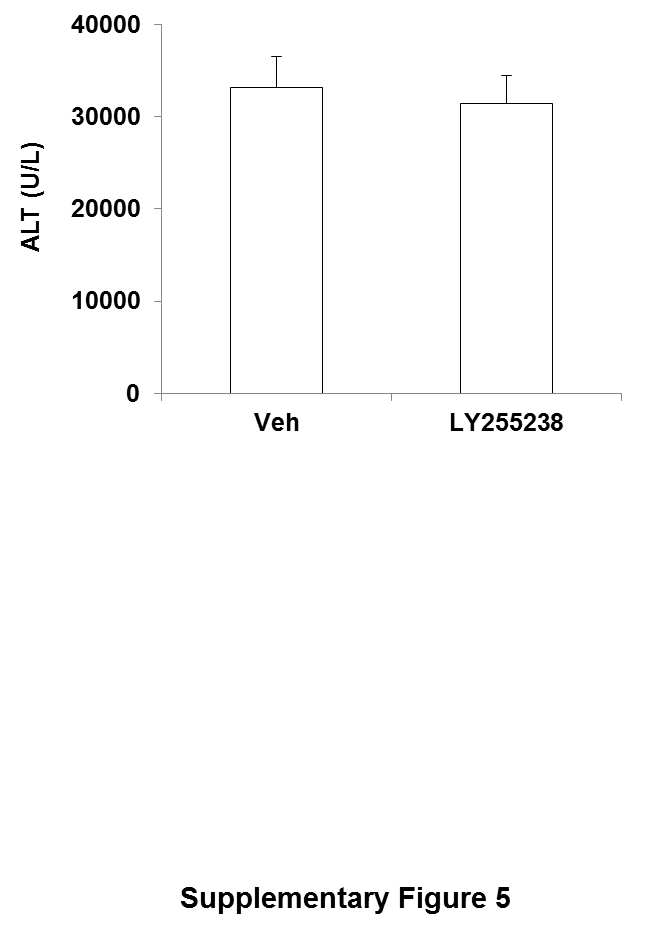

Supplement: Supplementary Information [file srep29650-s1.doc]
